# Draft genome sequence of *Cellulomonas carbonis* T26^T^ and comparative analysis of six *Cellulomonas* genomes

**DOI:** 10.1186/s40793-015-0096-8

**Published:** 2015-11-18

**Authors:** Weiping Zhuang, Shengzhe Zhang, Xian Xia, Gejiao Wang

**Affiliations:** State Key Laboratory of Agricultural Microbiology, College of Life Sciences and Technology, Huazhong Agricultural University, Wuhan, 430070 P. R. China

**Keywords:** *Cellulomonas*, *Cellulomonas carbonis*, Cellulolytic, Comparative genomics, Genome sequence

## Abstract

Most *Cellulomonas* strains are cellulolytic and this feature may be applied in straw degradation and bioremediation. In this study, *Cellulomonas carbonis* T26^T^, *Cellulomonas bogoriensis* DSM 16987^T^ and *Cellulomonas cellasea* 20108^T^ were sequenced. Here we described the draft genomic information of *C. carbonis* T26^T^ and compared it to the related *Cellulomonas* genomes. Strain T26^T^ has a 3,990,666 bp genome size with a G + C content of 73.4 %, containing 3418 protein-coding genes and 59 RNA genes. The results showed good correlation between the genotypes and the physiological phenotypes. The information are useful for the better application of the *Cellulomonas* strains.

**Electronic supplementary material:**

The online version of this article (doi:10.1186/s40793-015-0096-8) contains supplementary material, which is available to authorized users.

## Introduction

Strain T26^T^ (= CGMCC 1.10786^T^ = KCTC 19824 ^T^ = CCTCC AB2010450 ^T^) is the type strain of *Cellulomonas carbonis* which was isolated from coal mine soil [1]. The genus *Cellulomonas* was first proposed by Bergey et al. in 1923 [2]. To date, the genus *Cellulomonas* contains 27 species and mainly isolated from cellulose enriched environments such as soil, bark, wood and sugar field [1–4]. The common characteristics of the *Cellulomonas* strains are Gram-positive, rods, high G + C content (69–76 mol%) and cellulolytic, containing anteiso-C15:0 and C16:0 as the major fatty acids, and menaquinone-9(H4) as the predominant quinone. Most *Cellulomonas* strains can degrade cellulose and hemicellulose, making the strains applicable in paper, textile, and food industries, soil fertility and bioremediation [5–8]. The characterization of cellobiose phosphorylase, endo-1,4-xylanase, xylanases and endo-1,4-glucanase of *Cellulomonas* strains have been previously published [9–12].

So far, three genomes of *Cellulomonas* have been published including *Cellulomonas flavigena*DSM 20109^T^ [13], *Cellulomonas fimi*ATCC 484^T^ [14] and “*Cellulomonas gilvus”*ATCC 13127^T^^1^ [14] and showed a wide variety of cellulases and hemicellulases in their genomes [13, 14]. In order to provide more genomic information about *Cellulomonas* strains for potential industrial application, we sequenced the genomes of *Cellulomonas carbonis* T26^T^ [1], *Cellulomonas cellasea*DSM 20118^T^ [2] and *Cellulomonas bogoriensis*DSM 16987^T^ [15]. Here we present a summary genomic features of *C. carbonis* T26^T^ together with the comparison results of the six available *Cellulomonas* genomes.

## Organism information

### Classification and features

**Fig. 1:**
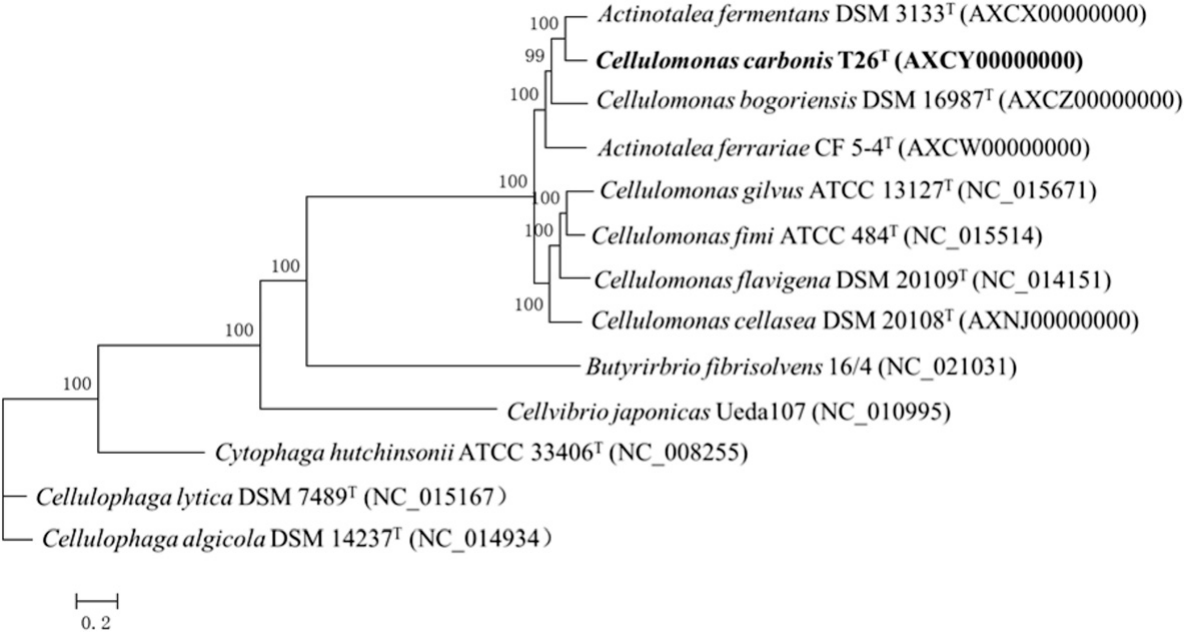
Phylogenetic tree showing the position of *C. carbonis* T26^T^ (shown in bold) based on aligned sequences of 105 single-copy conserved proteins shared among the 13 genomes. The conserved protein was acquired by OrthoMCL with a Match Cutoff 50 % and an E-value Exponent Cutoff 1-e5 [15, 16]. Phylogenetic analysis was performed using MEGA version 5.05 and the tree was built using the Maximum-Likelihood method [17] with 1000 bootstrap repetitions were computed to estimate the reliability of the tree. The corresponding GenBank accession numbers are displayed in parentheses

The taxonomic classification and general features of *C. carbonis* T26^T^ are presented in Table 1. A total of 105 single-copy conserved proteins were obtained within the 13 genomes by OrthoMCL with a Match Cutoff 50 % and an E-value Exponent Cutoff 1-e^5^ [16, 17]. Figure 1 shows the phylogenetic tree of *C. carbonis* T26^T^ and 12 related strains based on conserved gene sequences. The tree was constructed by MEGA 5.05 with Maximum-Likelihood method to determine phylogenetic position [18]. The genome based phylogenetic tree (Fig. 1) is similar to the 16S rRNA gene based phylogenetic tree [1].

**Table 1:** Classification and general features of *C. carbonis* T26^T^

MIGS ID	Property	Term	Evidence code^a^
	Classification	Domain *Bacteria*	TAS [33]
		Phylum *Actinobacteria*	TAS [34]
		Class *Actinobacteria*	TAS [35]
		Order *Micrococcales*	TAS [36]
		Family *Cellulomonadaceae*	TAS [37]
		Genus *Cellulomonas*	TAS [1, 38]
		Species *Cellulomonas carbonis*	TAS [1]
		(Type) strain: T26^T^ = (CGMCC 1.10786^T^ = KCTC 19824^T^ = CCTCC AB2010450^T^)	
	Gram stain	Positive	TAS [1]
	Cell shape	Rod-shaped	TAS [1]
	Motility	Motile	TAS [1]
	Sporulation	Non-sporulating	NAS
	Temperature range	4-45 °C	TAS [1]
	Optimum temperature	28 °C	TAS [1]
	pH range; Optimum	6-10;7	TAS [1]
	Carbon source	D-glucose, L-arabinose, mannose, N-acetyl	TAS [1]
		glucosamine, maltose, gluconate, sucrose, glycogen, salicin, D-melibiose, D-sorbitol, xylose, D-lactose, D-galactose, D-fructose, and raffinose.	
MIGS-6	Habitat	Soil	TAS [1]
MIGS-6.3	Salinity	0-7 % NaCl (*w/v*)	TAS [1]
MIGS-22	Oxygen requirement	Aerobic	TAS [1]
MIGS-15	Biotic relationship	free-living	TAS [1]
MIGS-14	Pathogenicity	non-pathogen	NAS
MIGS-4	Geographic location	Tianjin city,China	TAS [1]
MIGS-5	Sample collection	2012	TAS [1]
MIGS-4.1	Latitude	39°01'49.77" N	TAS [1]
MIGS-4.2	Longitude	117°11'20.20" E	TAS [1]
MIGS-4.4	Altitude	Not reported	TAS [1]

^a^Evidence codes - *IDA* Inferred from Direct Assay, *TAS* Traceable Author Statement (i.e., a direct report exists in the literature), *NAS* Non-traceable Author Statement (i.e., not directly observed for the living, isolated sample, but based on a generally accepted property for the species, or anecdotal evidence). These evidence codes are from the Gene Ontology project [23]

**Fig. 2:**
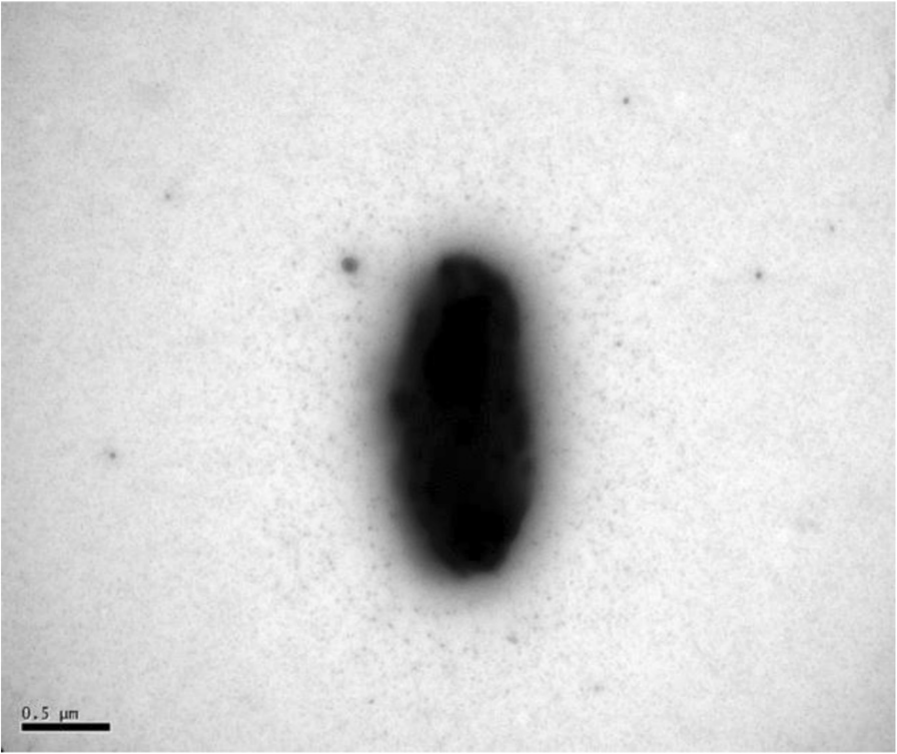
A transmission electron micrograph of strain T26^T^ grown on LB agar at 28 °C for 48 h. The bar indicates 0.5 μm

Strain *C. carbonis* T26^T^ is Gram-positive, aerobic, motile and rod-shaped (0.5–0.8 × 2.0–2.4 μm) (Fig. 2). The colonies are yellow-white, convex, circular, smooth, non-transparent and about 1 mm in diameter after 3 days incubation on R2A agar at 28 °C [1]. The optimal growth occurs at 28 °C (Table 1). The strain was able to hydrolyse CM-cellulose, starch, gelatin, aesculin and positive in catalase and nitrate reduction [1]. *C. carbonis* T26^T^ was capable of utilizing a wide range of sole carbon sources including D-glucose, L-arabinose, mannose, N-acetyl glucosamine, maltose, gluconate, sucrose, glycogen, salicin, D-melibiose, D-sorbitol, xylose, D-lactose, D-galactose, D-fructose and raffinose [1, Table 1].

#### Chemotaxonomy

*C. carbonis* T26^T^ contains anteiso-C_15:0_ (33.6 %), anteiso-C_15:1_ A (22.1 %), C_16:0_ (14.4 %) and C_14:0_ (12.1 %) as the major fatty acids and menaquinone-9(H4) as the predominant respiratory quinone. The major polar lipids of this strain were diphosphatidylglycerol and phosphatidylglycerol [1].

## Genome sequencing information

### Genome project history

This organism was selected for sequencing particularly due to its cellulolytic activity and other applications. Genome sequencing was performed by Majorbio Bio-pharm Technology in April-June, 2013. The raw reads were assembled by SOAPdenovo v1.05. The genome annotation was performed at the RAST server version 2.0 [19] and the NCBI Prokaryotic Genome Annotation Pipeline and has been deposited at DDBJ/EMBL/GenBank under accession number AXCY00000000. The version described in this study is the first version AXCY01000000. The project information are summarized in Table 2.

**Table 2:** Project information

MIGS ID	Property	Term
MIGS-31	Finishing quality	Draft
MIGS-28	Libraries used	Illumina Paired-End library (300 bp insert size)
MIGS-29	Sequencing platforms	Illumina Miseq 2000
MIGS-31.2	Fold coverage	343.5×
MIGS-30	Assemblers	SOAPdenovo v1.05
MIGS-32	Gene calling method	GeneMarkS+
	Locus tag	N868
	GenBank ID	AXCY00000000
	GenBank Date of Release	October 17, 2014
	GOLD ID	Gi0055591
	BIOPROJECT	PRJN215138
MIGS-13	Source material identifier	T26^T^
	Project relevance	Genome comparison

### Growth conditions and genomic DNA preparation

Strain *C. carbonis* T26^T^ was grown aerobically in 50 ml LB medium at 28 °C for 36 h with 160 rpm shaking. Cells were collected by centrifugation and about 20 mg pellet was obtained. Genomic DNA was extracted, concentrated and purified using the QiAamp kit (Qiagen, Germany). The quality of DNA was assessed by 1 % agarose gel electrophoresis and the quantity of DNA was measured using NanoDrop Spectrophotometer 2000 (Equl-Thermo SCIENTIFIC, USA). About 8.8 μg of genomic DNA was sent to Shanghai Majorbio Bio-pharm Technology Co., Ltd for library preparation and sequencing.

### Genome sequencing and assembly

The genome of *C. carbonis* T26^T^ was sequenced by Illumina Hisep2000 pair-end technology at Shanghai Majorbio Bio-pharm Technology Co., Ltd. A 300 bp Illumina standard shotgun library was constructed and generated 7,703,453 × 2 reads totaling 1,556,097,506 bp Illumina data. Raw reads were filtered using the FastQC toolkit and optimizing through local gap filling and base correction with Gap Closer. All general aspects of library construction and sequencing can be found at the Illumina’s official website [20]. Using SOAPdenovo v1.05 version [21], 7,324,578 × 2 paired reads and 349,082 single reads were assembled *de novo*. Due to very high GC content, the final draft assembly yield 547 contigs arranged in 414 scaffolds with 343.5 × coverage. The final assembly results showed that 97.6 % of the bases present in larger contigs (>1000 bp), and the contig N50 is 29,777 bp. The draft genome of *C. carbonis* T26^T^ is present as a set of contigs ordered against the complete genome of *C. flavigena*DSM 20109^T^ using Mauve software [22].

### Genome annotation

The draft genome sequence of *C. carbonis* T26^T^ was annotation through the RAST server version 2.0 and the National Center for Biotechnology Information Prokaryotic Genome Annotation Pipeline. Genes were identified using the gene caller GeneMarkS^+^ with the similarity-based gene detection approach [23]. The predicted CDSs were translated and used to search the NCBI Nonredundant Database, Pfam [24], KEGG [25], and the NCBI Conserved Domain Database through the Batch web CD-Search tool [26]. The miscellaneous features were prediction by WebMGA [27], TMHMM [28] and SignalP [29]. The putative cellulose-degrading enzymes were identified through Carbohydrate-Active enZYmes Database (CAZymes) Database [30].

## Genome properties

**Fig. 3:**
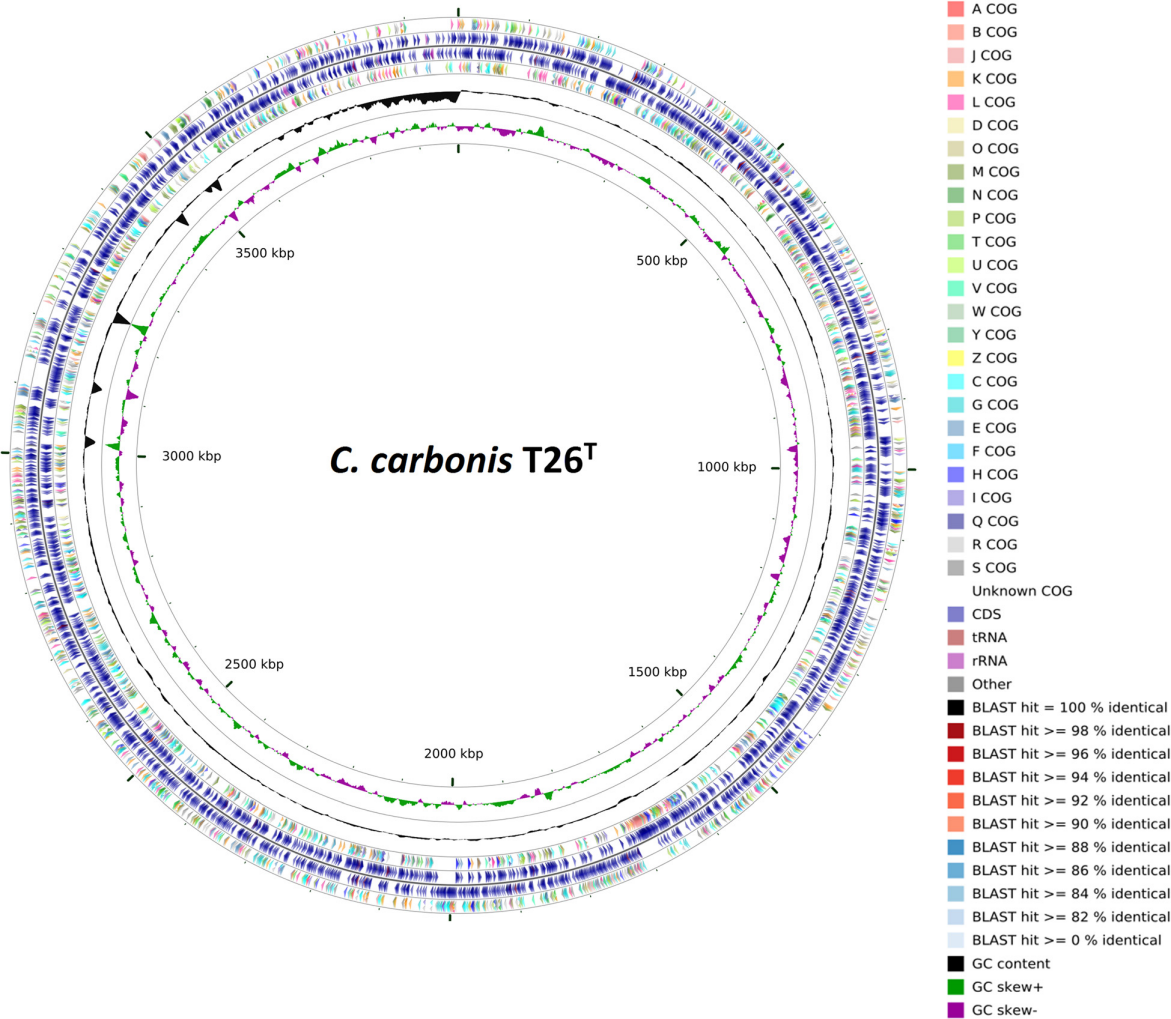
A graphical circular map of the *C. carbonis* T26^T^ genome performed with CGview comparison tool [39]. From outside to center, ring 1, 4 show protein-coding genes colored by COG categories on forward/reverse strand; ring 2, 3 denote genes on forward/reverse strand; ring 5 shows G + C% content plot, and the innermost ring shows GC skew

The whole genome of *C. carbonis* T26^T^ is 3,990,666 bp in length, with an average GC content of 73.4 %, and comprised of 547 contigs. The genome properties and statistics are summarized in Table 3 and Fig. 3. From a total of 3513 genes, 3418 protein-coding genes were identified and 71 % of them were assigned putative functions, while the remainder was annotated as hypothetical proteins. In addition, 36 pseudogenes, 11 rRNA, 46 tRNAs and 1 ncRNA were identified. The distributions of genes among the COGs functional categories are shown in Table 4.

**Table 3:** Genome statistics

Attribute	Value	% of total^a^
Genome size (bp)	3,990,666	100.00
DNA coding (bp)	2,927,153	73.35
DNA G + C (bp)	3,368,220	84.40
DNA scaffolds	414	100.00
Total genes	3513	100.00
Protein-coding genes	3418	97.30
RNA genes	59	1.68
Pseudo genes	36	1.02
Genes in internal clusters	1435	40.85
Genes with function prediction	2481	71.00
Genes assigned to COGs	1450	41.28
Genes with Pfam domains	2231	63.51
Genes with signal peptides	253	7.20
Genes with transmembrane helices	764	21.75
CRISPR repeats	0	-

^a^The total is based on either the size of the genome in base pairs or the total number of protein coding genes in the annotated genome

**Table 4:** Number of genes associated with general COG functional categories

Code	Value	%age^a^	Description
J	152	4.45	Translation, ribosomal structure and biogenesis
A	4	0.12	RNA processing and modification
K	244	7.14	Transcription
L	136	3.98	Replication, recombination and repair
B	1	0.03	Chromatin structure and dynamics
D	29	0.85	Cell cycle control, Cell division, chromosome partitioning
V	58	1.70	Defense mechanisms
T	195	5.71	Signal transduction mechanisms
M	141	4.13	Cell wall/membrane biogenesis
N	54	1.58	Cell motility
U	61	1.78	Intracellular trafficking and secretion
O	106	3.10	Posttranslational modification, protein turnover, chaperones
C	181	5.30	Energy production and conversion
G	298	8.72	Carbohydrate transport and metabolism
E	198	5.79	Amino acid transport and metabolism
F	72	2.11	Nucleotide transport and metabolism
H	116	3.39	Coenzyme transport and metabolism
I	91	2.66	Lipid transport and metabolism
P	130	3.80	Inorganic ion transport and metabolism
Q	48	1.40	Secondary metabolites biosynthesis, transport and catabolism
R	340	9.95	General function prediction only
S	199	5.82	Function unknown
-	1968	57.58	Not in COGs

^a^The percentage is based on the total number of protein-coding genes in the annotated genome

## Insights from the genome sequence

**Fig. 4:**
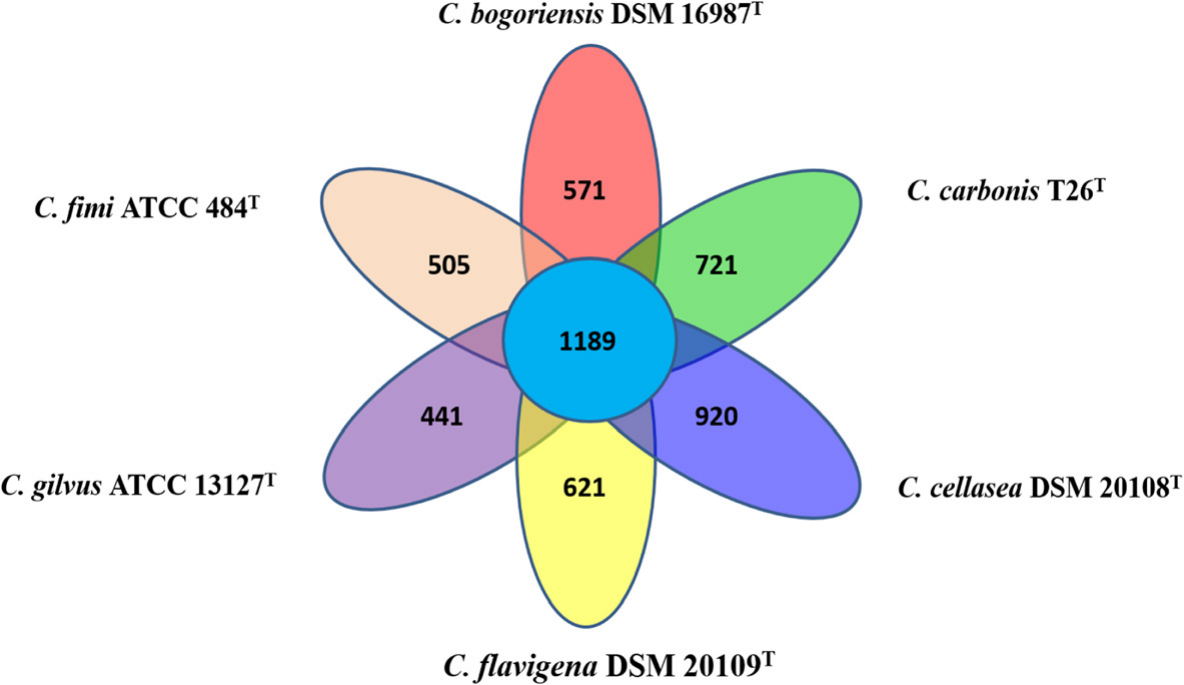
Ortholog analysis of the six *Cellulomonas* genomes conducted using OrthoMCL. The total numbers of shared proteins among the six genomes and unique proteins from each species were tabulated and presented as a Venn diagram

**Fig. 5:**
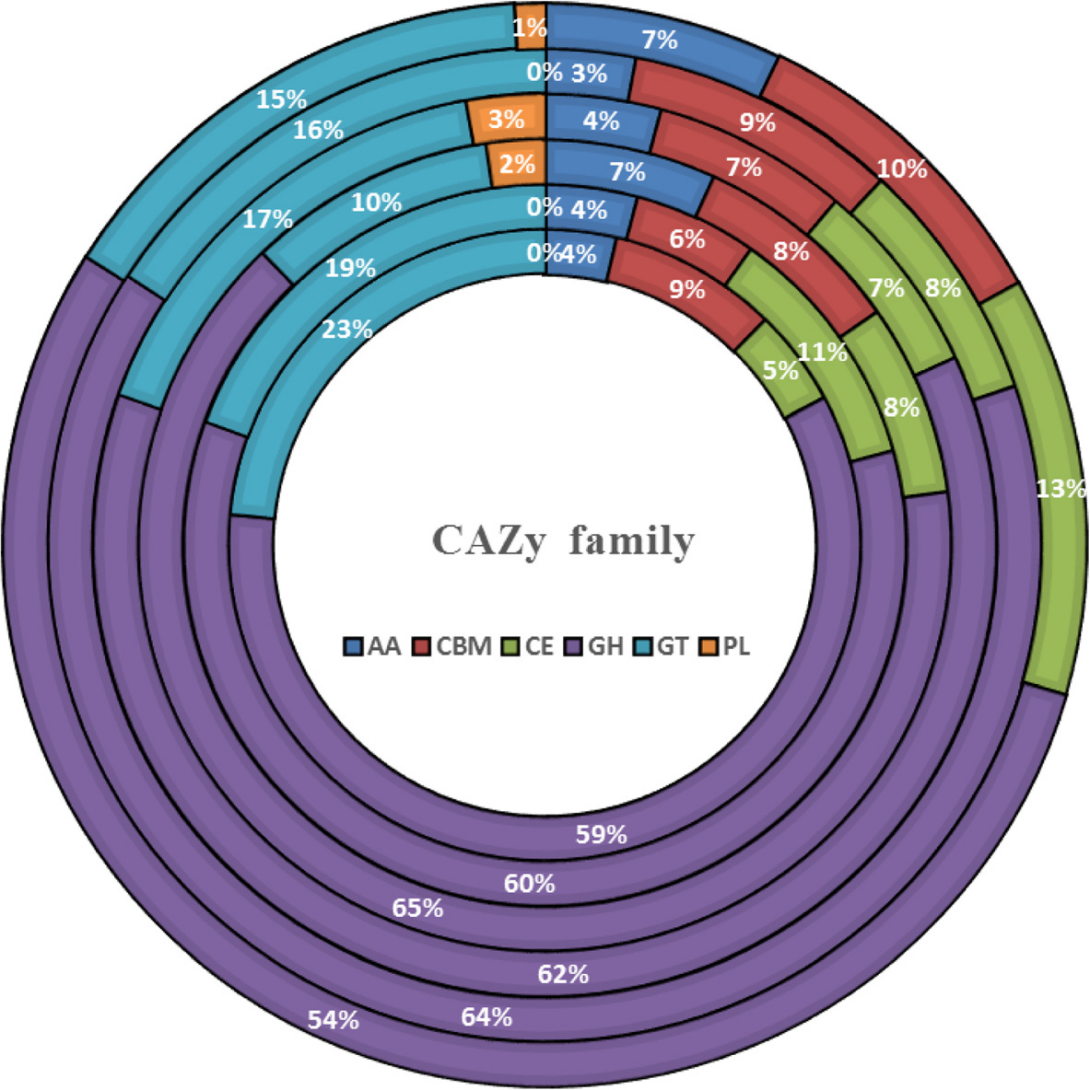
Comparative analysis of putative proteins of CAZy family of six *Cellulomonas* genomes. From outside to center, ring 1 is *C. flavigena* DSM 20109^T^; ring 2 is *C. gilvus* ATCC 13127^T^; ring 3 is *C. fimi* ATCC 484^T^; ring 4 is *C. cellasea* DSM 20108^T^; ring 5 is *C. bogoriensis* DSM 16987^T^; ring 6 is *C. carbonis* T26^T^. AA, auxiliary activities; CBM, carbohydrate binding module; CE, carbohydrate esterase; GH, glycoside hydrolases; GT, glycosyltransferase; PL, polysaccharide lyase

In order to reveal more genomic information for better application of the *Cellulomonas* strains, the genomic features of *C. carbonis* T26^T^ together with the comparison results of the six *Cellulomonas* genomes were analyzed (Table 5). OrthoMCL analysis with a Match cutoff of 50 % and an E-value Exponent cutoff of 1-e5 identified 1189 single-copy conserved proteins among the six *Cellulomonas* genomes (Fig. 4). Several carbohydrate-active enzymes have been identified and classified into different families of glycoside hydrolases, carbohydrate binding modules, carbohydrate esterases, auxiliary activities and polysaccharide lyases [31] (Fig. 5, Additional file 1: Table S1). Some putative glycoside hydrolases may be responsible for the ability of *Cellulomonas* spp. to utilize various sole carbon sources.

**Table 5:** General features of the six *Cellulomonas* genomes

Strain	Isolation source	Genome size (Mb)	Coverge	CDSs	RNA	G + C content	GenBank No.
*C. gilvus* ATCC 13127^T^	feces	3.53	-	3164	54	73.8 %	NC_015671
*C. fimi* ATCC 484^T^	soil	4.27	-	3761	54	74.7 %	NC_015514
*C. flavigena* DSM 20109^T^	soil	4.12	-	3678	54	74.3 %	NC_014151
*C. bogoriensis* DSM 16987^T^	sediment and water	3.19	368.2 x	2898	51	72.2 %	AXCZ00000000
*C. carbonis* T26^T^	coal mine soil	3.99	343.5 x	3418	59	73.3 %	AXCY00000000
*C. cellasea* DSM 20108^T^	NR	4.66	724.0 x	3560	44	74.6 %	AXNJ00000000

**Fig. 6:**
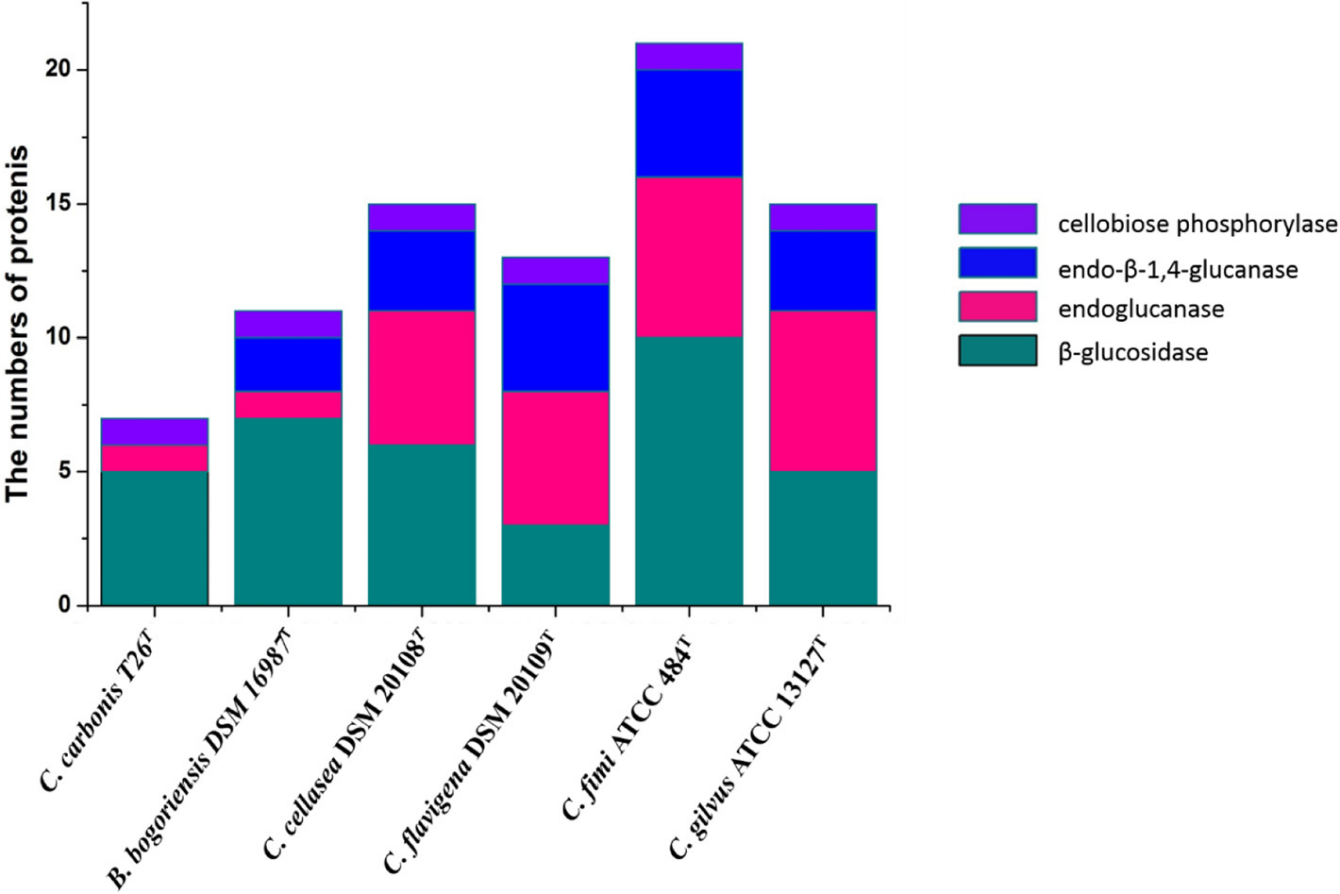
The distribution of cellulases in six *Cellulomonas* genomes. The cellulases are β-glucosidase, endoglucanase, endo-β-1,4-glucanase and cellobiose phosphorylase

Some potential cellulose-degrading enzymes were found and analyzed (Fig. 6, Additional file 1: Table S2). *C. fimi*ATCC 484^T^ possesses the highest number of putative cellulases, including ten members of β-glucosidases (GH1 and GH3); six members of endoglucanases (GH6 and GH9); four endo-β-1,4-glucanases (GH48 and GH5) and one cellobiose phosphorylase (GH94). *C. carbonis* T26^T^ has the fewest putative cellulases, including one cellobiose phosphorylase (GH94); one endoglucanase (GH6) and five β-glucosidases (GH1 and GH3). Cellulose activity assays were performed on Congo-Red agar media [32] and all of the six *Cellulomonas* strains yielded a cellulose clearing zone on the media (data not shown). The Kyoto Encyclopedia of Genes and Genomes was used to construct metabolic pathways and all of the six *Cellulomonas* strains have the complete cellulose degradation pathways (data not shown).

In addition to the utilization of cellulose, the *Cellulomonas* strains are also known to degrade hemicelluloses. A large number of putative intracellular and extracellular xylan degrading enzymes have been identified in the *Cellulomonas* genomes, such as endo-1-4,-β-xylanase, β-xylosidase, α-L-arabinofuranosidase, acetylxylan esterase and α-glucuronidase (Additional file 1: Table S3) which suggests the capacity to degrade hemicelluloses. We also found a large number of α-amylases which are responsible to the degradation of starch in the six *Cellulomonas* genomes (Additional file 1: Table S4) suggest the potential application in bioremediation of food industrial wastewater.

## Conclusions

The genomic information of *C. carbonis* T26^T^ and the comparison results of the six *Cellulomonas* genomes revealed a high degree of putative cellulases, hemicellulases. In addition, we found that the genomes also contain members of α-amylases. These information provides a genomic basis for the better application of *Cellulomonas* spp. in industry and environmental bioremediation. In addition, the genomes possess many putative carbohydrate-active enzymes which is in agreement with their physiological ability to utilize various sole carbon sources.

## Additional file

Additional file 1: Table S1. Putative CAZy family and locus_tag number in the six *cellulomonas* genomes. **Table S2.** Putative cellulases in the six cellulomonas genomes. **Table S3.** Putative hemicellulases in the six cellulomonas genomes**. Table S4.** Putative amylases in the six cellulomonas genomes. (XLSX 29 kb)

## Abbreviations

RAST: Rapid Annotation using Subsystem Technology
PGAP: Prokaryotic Genome Annotation Pipeline

## Footnotes

1 Editorial note – although designated as a type strain of *Cellulomonas gilvus* by Christopherson et al., this strain continues to be listed as a non-type strain of *Cellvibrio gilvus* in the ATCC catalogue. At present, neither name has standing in the taxonomic literature.
